# Effects of Calcium Soaps from Palm, Canola and Safflower Oils on Dry Matter Intake, Nutrient Digestibility, Milk Production, and Milk Composition in Dairy Goats

**DOI:** 10.3390/ani10101728

**Published:** 2020-09-23

**Authors:** Einar Vargas-Bello-Pérez, Lizbeth Esmeralda Robles-Jimenez, Rafael Ayala-Hernández, Jose Romero-Bernal, Nazario Pescador-Salas, Octavio Alonso Castelán-Ortega, Manuel González-Ronquillo

**Affiliations:** 1 Department of Veterinary and Animal Sciences, Faculty of Health and Medical Sciences, University of Copenhagen, Grønnegårdsvej 3, DK-1870 Frederiksberg C, Denmark; 2 Facultad de Medicina Veterinaria y Zootecnia, Universidad Autónoma del Estado de México, Instituto Literario 100, CP 50000 Toluca, Mexico; lizroblez@hotmail.com (L.E.R.-J.); elpipilaenmadison@yahoo.com (R.A.-H.); gazapo79@yahoo.com.mx (J.R.-B.); npescadors@uaemex.mx (N.P.-S.); oacastelano@uaemex.mx (O.A.C.-O.)

**Keywords:** calcium soaps, goats, canola oil, safflower oil, nutrient digestibility

## Abstract

**Simple Summary:**

Dietary fats can increase energy density in dairy goat diets. However, dietary fats are subject to changes in rumen that affect nutrient intake and digestibility and milk production. In order to by-pass rumen degradation, one strategy is to protect those fats by saponification, which results in calcium soaps of fatty acids. Thus, this study determined the effect of calcium soaps of either palm (PO), canola (CO) or safflower (SO) oils on dry matter intake, digestibility and milk production in dairy goats. Compared with PO and CO, milk production increased with SO, while CO was more digestible. Compared to calcium soaps from PO, SO resulted in increased milk yield without negative effects on digestibility and nutrient intake. Overall, compared with the traditional use of calcium soaps manufactured from PO, at an inclusion of 2.7% dry matter, calcium soaps of SO can be used in goat diets to increase milk production, milk protein and milk fat yields without negative effects on nutrient intake and digestibility.

**Abstract:**

This study determined the effect of protected dietary oils on dry matter intake (DMI), digestibility and milk production in dairy goats. Nine Saanen goats were used in a 3 × 3 Latin square design with three periods of 25 days. A basal diet based on barley hay and corn silage was supplemented with 2.7% DM of calcium soaps of either palm (PO), canola (CO) or safflower (SO) oils. Data for dry matter intake, nutrient digestibility and milk production was analyzed using the general linear model (GLM) procedure of SAS. Gas production data was analyzed using the procedure of non-linear regression analysis (PROC NLIN) from SAS. Nutrient intakes were not affected by treatments. However, compared with CO, the digestibility of dry matter (653 vs. 552 and 588 g/kg), organic matter (663 vs. 559 and 606 g/kg) and neutral detergent fiber (616 vs. 460 and 510 g/kg) were lowered (*p* < 0.001) by SO and PO. Compared with CO, in vitro gas production increased (*p* < 0.001) in PO and SO (174 vs. 201 and 206 mL gas/g incubated DM). Compared with PO and CO, milk production increased (*p* < 0.001) with SO (0.88 and 0.95 vs. 1.10 kg/d, respectively). With regard to PO and SO, CO decreased fat (34 and 35 vs. 32 g/d) and protein (35 and 38 vs. 30 g/d) in milk. In conclusion, compared to the traditional use of calcium soaps manufactured from PO, protected SO resulted in increased milk yield without negative effects on digestibility and nutrient intake.

## 1. Introduction

The use of different types and levels of fat in the diet has major effects on the quantity and composition of milk from goats and sheep [1]. The palm oil (PO) industry produces several by-products (i.e., palm oil, palm press fiber, palm kernel cake and palm oil decanter cake) that represent alternatives for feeding ruminants [2]. Their inclusion in diets can be an effective measure in overcoming the lack of grazing pasture for goats and sheep. On the other hand, in the past decade, the production of canola oil (CO) has increased, and is available at a reasonable cost. CO by-products, such as crude canola oil, can thus provide an alternative to the use of PO in small ruminants [3]. In fact, the blend of PO and CO has been reported to increase polyunsaturated fatty acids (PUFA) in muscle without having detrimental effects on nutrient intake and digestibility and growth performance in goats [4].

In dairy goats, adding safflower oil (SO) at 50 g/kg dry matter (DM) to goat diets based on chopped alfalfa hay has been shown to be an effective strategy to increase milk PUFA without detrimental effects on feed intake and milk production [5]. The intake of lipids in the form of protected fats allows the increase of fatty acids (FA) available for intestinal absorption [6], without causing negative effects on the ruminal microbial population and feed digestibility [7]. The use of various lipid supplements in goats, such as calcium soaps, can increase milk production, modify the percentage of milk fat and produce changes in the milk FA profile [8].

Calcium soaps based on different vegetable oils have been used in ruminant production; for example, feeding calcium soaps of palm oil has been shown to increase milk fat in early lactating ewes [9,10] and increase feed conversion ratio without affecting carcass yield in growing lambs [11]. On the other hand, calcium soaps from canola, soybean and linseed oils have been used to increase milk PUFA in cows [12]. In ewes, safflower oil has been used as a feeding strategy to improve milk production and increase the contents of PUFA in milk [13]. However, information on calcium soaps of safflower oil in goat diets is not available.

It is known that in small ruminants, the degree of FA saturation of lipid sources will influence animal performance [3], milk composition [14] and milk FA profile [15,16]. In fact, in goat mammary epithelial cells, saturated and unsaturated long-chain FAs exert differential expression of genes related to lipid metabolism [17]. To our knowledge, no research has been done to study the concurrent benefits of feeding calcium soaps of CO or SO on nutrient digestibility and productive traits in dairy goats, as opposed to the more traditional use of calcium soaps manufactured from PO [18]. Thus, the objective of the present study was to evaluate three sources of protected oils (calcium soaps) as an energy supplement in dairy goats and to assess the effects of protected dietary oils on dry matter intake, nutrient digestibility, milk production and milk composition in dairy goats. To this end, calcium soaps of either palm oil, canola oil or safflower oil were used. The hypothesis of this study was that nutrient digestibility, milk production and milk composition are affected differentially by the degree of FA saturation of dietary lipids, with palm oil being a saturated FA source and canola and safflower oils being unsaturated FA sources. In this study, the reference condition (control diet) was calcium soap from palm oil, as it is the most common protected oil available in the Mexican market.

## 2. Materials and Methods

### 2.1. Animals, Experimental Design and Diets

The present study was carried out in the Animal Science farm of the School of Veterinary Medicine and Animal Science of the Universidad Autónoma del Estado de México. All animal studies were conducted according to the Animal Care and Use Committee of Veterinary and Animal Science from Universidad Autónoma del Estado de México (Toluca, Mexico) (Project ID UAEMex 3060-2011).

Nine Saanen goats [51 ± 0.4 kg of body weight; 19.1 ± 0.02 metabolic live weight (LW^0.75^)] were used in a 3 × 3 Latin square design with three periods of 25 days consisting of 20 days for adaptation followed by 5 days of sampling. At the beginning of the study, all animals had 160 d of lactation. A basal diet based on barley hay and corn silage was supplemented with 2.7% DM of calcium soaps of either palm (PO; Megalac-R^®^, Arm & Hammer Animal Nutrition, Princenton, NJ, USA), canola (CO) or safflower (SO) oils. Calcium salts of PO were considered as the reference condition (control). The chemical composition of individual feedstuffs and lipid supplements used in the experimental diets is shown in Table 1.

The basal diet consisted of forage (barley hay and corn silage) and concentrate (cracked corn grain and soybean meal) supplemented with vitamins and minerals (Multitec of Malta^®^, Celaya Guanajuato, Mexico). Dietary treatments (Table 2) were formulated to be iso-proteic and iso-energetic (111 g CP/d and 12 MJ ME/d) [19]. Diets were offered as total mixed rations and calcium soaps of PO, CO and SO were mixed manually at each individual feed bunk. Animals were kept in individual metabolic cages (1.20 × 0.80 m) with free access to water. Dietary treatments were divided into two meals and were offered daily at 08:00 and 16:00 h.

Feed intake, amount of feces and urine were recorded daily, but only measurements collected during the last five days of each experimental period were used for statistical analysis. Individual samples of feed, feces and urine were collected at 08:00 h during the last five days of each experimental period and then frozen at −20 °C until further analysis. For feces and urine, only 10% of the total sample collected was used for analysis.

Feces and urine samples were used to determine N intake and excretion. Urine N was analyzed using micro-Kjeldahl analysis. For feces, dry matter (DM; 930.15), organic matter (OM; 942.05) and nitrogen (N; 990.02) were determined using the Association of Official Analytical Chemists [20] methods, while neutral detergent fiber (NDF) and acid detergent fiber (ADF) were determined following Van Soest et al. [21] methods with alpha amylase and uncorrected ash content. Individual body weights (BW) were measured at the beginning and end of each experimental period.

### 2.2. Calcium Soap Elaboration

Oils used for calcium soap elaboration had specific FA contents: palm oil was composed of 65 g/100 g of palmitic acid, canola oil was composed of 65 g/100 g of oleic acid and safflower oil was composed of 65 g/100 g of linoleic acid.

The saponification process for canola and safflower oils was performed using the Jenkins and Palmquist [22] double decomposition method. Briefly, for each 100 g of oil, 25 g of calcium chloride, 5 g of calcium carbonate, 30 mL of 96% alcohol, 30 mL of 30% sodium hydroxide and distilled water were used. At the end of the saponification process, the soaps were introduced at 60 °C for 48 h of dehydration [23]. The soaps were then ground to 1 mm in diameter and used in the experimental diets.

### 2.3. Chemical Composition of Diets and Milk

To determine the dry matter (DM) content in feed, refusals and feces, samples were dried in a forced-air oven at 60 °C for 48 h, and subsequently ground in a Wiley mill using a 3 mm screen (Arthur H. Thomas, Philadelphia). For experimental diets, organic matter (OM) was determined by incineration at 550 °C for 3 h. Standard procedures [24] were used to determine the DM (934.01), Kjeldahl N (984.13), and ether extract (920.39). Neutral detergent fiber (NDF) and acid detergent fiber (ADF) were analyzed according to Van Soest et al. [21], including α-amylase and sodium-sulfite for NDF analysis using the ANKOM system (ANKOM Technology, Macedon, NY,) for NDF and ADF.

Milk yield was recorded on the last 5 days of each experimental period, and was collected at 08.00 h using a volumetric milk meter and considered for statistical analysis. Samples were preserved with potassium dichromate and analyzed for protein, fat, lactose, total solids (TS) and non-fat solids (NFS) using a MilkoScan 133B (Foss Electric, Hillerød, Denmark).

### 2.4. In Vitro Gas Production

The in vitro gas production technique using a pressure transducer [25] was used to determine the kinetics of rumen fermentation. Three rumen cannulated lactating goats (40 ± 3 kg of body weight) were used as donors of rumen fluid and fed with the same experimental basal diet. Equal amounts (150 mL) of rumen fluid were collected and filtered through four layers of cheesecloth.

The buffer solution was prepared according to Menke and Steingass [26], where 0.800 g DM of each ingredient and each diet mixture were incubated in glass bottles of 125 mL. The bottles were filled under anaerobic conditions with 10 mL of rumen inoculum and 90 mL of an incubation solution in 1 L. This solution consisted of 238 mL/L of buffer solution (14 g NaHCO_3_ and 1.5 g (NH_4_)HCO_3_ per L), 238 mL/L of a macro mineral solution (5.7 g Na_2_HPO_4_, 6.2 g KH_2_PO_4_ and 0.6 g MgSO_4._7H_2_O per L), 474 mL/L of distilled water, 0.1 mL/L of micro minerals (13.2 g CuCl_22_H_2_O, 10.0 g MnCl_24_H_2_O, 1.0 g CoCl_26_H_2_O, 8.0 g FeCl_26_H_2_O and made up to 100 mL with H_2_O) and 50 mL/L of a reduction solution (47.5 mL distilled water, 2 mL of 1N NaOH and 313 mg HCl-cysteine), and resazurin (phenoxazine dye). Two additional bottles without substrate were also prepared as blanks in order to adjust for the potential contribution of other soluble extracts on overall gas production and to correct readings of substrate, including bottles from the self-fermentation of rumen inocula.

Bottles were filled with the incubation solution under a CO_2_ stream, sealed and incubated for 96 h in a water bath at 39 °C. The gas volume was recorded at 3, 6, 9, 12, 24, 36, 48, 72 and 96 h of incubation in three series of incubation. The pressure produced on each bottle was measured with an HD8804 manometer provided with a TP804 pressure gauge (Delta OHM, Caselle di Selvazzano, Italy). Readings corrected for atmospheric pressure were converted to volume (mL) using a pre-established linear regression [mL gas = (2.7384x) − 0.0243, n = 45, R^2^ = 0.994, where x = psi/hour] and expressed as a unit of incubated DM.

The kinetic parameters for gas production (GP) were estimated through an iterative procedure of non-linear regression analysis (PROC NLIN) with SAS [27] according to Krishnamoorthy et al. [28] (1991) and calculated as:

GP = B (1-e^-Ct^) (1)

where GP = gas production (mL gas/g^−1^ initial DM), B = total gas production (mL gas g^−1^ initial DM), C = rate of degradation with respect to time (hours) and *t* = time (h).

### 2.5. Calculations

Nutrient digestibility (g/kg) was determined as [(nutrient intake, g/d – nutrient excreted, g/d)/(nutrient intake, g/d)] × 1000. Fat-corrected milk at 3.5% (FCM) was calculated as FCM (kg/d) = [milk (kg/d) × 0.432] + [fat kg/d) × 16.216]. Energy corrected milk (ECM) was calculated as ECM = [milk (kg/d) × 0.327] + [fat (kg/d) × 12.86] + [protein (kg/d) × 7.65] [29]. Feed efficiency (FE) was calculated as FE = milk yield (kg/d)/dry matter intake (kg/d). Adjusted FE was = 3.5% FCM (kg)/DM intake (kg/d) and milk production efficiency (MPE) was = kg of milk produced/kg of crude protein ingested.

After 96 h of incubation, the accumulated gas was released, and the fermentation residues from each flask were dried at 60 °C for 48 h to calculate the dry matter disappearance: DMD (mg/100 mg) = (DM disappeared 96 h)/(DM initial) × 100, where: DMD (mg/g DM disappeared at 96 h), DM initial (mg/g DM). The partitioning factor at 96 h of incubation (PF96—a measure of fermentation efficiency) was calculated as the ratio of in vitro DMD (DMD, mg/100 mg) to the volume (mL) of GP at 96 h (i.e., DMD/total gas production (GP96)) according to Blümmel et al. [30]. Gas yield (GY24) was calculated as the volume of gas (mL gas/g DM) produced after 24 h of incubation divided by the amount of DMD (g) as: gas yield (GY24) = (mL gas 24 h/g DM)/g DMD. Short-chain fatty acid (SCFA) concentrations were calculated according to Getachew et al. [31] (2002) as: SCFA (mmol/200 mg DM) = 0.0222 GP—0.00425. Where GP was the 24 h net gas production (mL/200 mg DM). Microbial crude protein (MCP) biomass production was calculated according to Blümmel et al. [30] as: MCP (mg/g DM) = mg DMD − (mL gas × 2.2 mg/mL), where 2.2 mg/mL was a stoichiometric factor that expressed mg of C, H and O required for the production of SCFA gas associated with the production of 1 mL of gas.

### 2.6. Statistical Analysis

The in vivo data was subjected to analysis of variance using the general linear model (GLM) procedure of SAS [27] in a replicated (n = 3) 3 × 3 Latin square design.

Y_ijk_ = µ + P_i_ + A_j_ + T_(k)_ + E_ijk_ (2)

where Y_ijk_ = response variable in period i, animal j, treatment k, and µ = overall mean, P_i_ = effect of period i, A_j_ = effect of the animal, T_(k)_ = effect of treatment and E_ijk_ = random error. The fixed effects were the experimental period and treatment, and the random effect was the individual goat. Least square means with their standard errors were reported, and treatment effects were declared significant at *p* < 0.05. The in vitro gas production data were subjected to analysis of variance (ANOVA) using the general linear model (PROC GLM) from SAS [27]. The Tukey test (*p* < 0.05) was used to interpret any significant difference between the mean values. Mean differences were considered significant at *p* < 0.05 and *p* < 0.001. Standard errors of means were calculated from the residual mean square in the analysis of variance.

## 3. Results and Discussion

Supplementing different types of dietary fat can be a useful strategy to increase the energy supply for lactating animals without resorting to cereals, which can result in costly diets. Therefore, in the present study, the choice was to use a modest amount of dietary fat supplementation (as rumen-protected fats), which was enough to supply adequate amounts of dietary energy without compromising productive traits in lactating goats.

### 3.1. Ingredients and Chemical Composition of Dietary Treatments

Contents of DM, crude protein (CP), NDF and ADF in barley hay, corn silage and corn grain agreed with previous reports [19]. Except for ether extract (EE), dietary treatments did not change in their chemical composition because all treatments had the same basal diet, which was formulated to be iso-proteic and iso-energetic.

### 3.2. Nutrient Intake and Digestibility

Metabolic live weight was similar among treatments (Table 3). Data related to the effects of lipid supplementation on body weight changes in lactating goats are limited, and responses are usually variable between protected and unprotected vegetable oil sources [32]. In this study, the observed changes were relatively small, showing that animals were, on average, under a positive energy balance, and this was observed with body-weight gains at the end of the experimental trial.

Appropriate nutrient intake and digestibility are key factors influencing productive traits in lactating animals. These factors determine the profitability of animal production systems. Nutrient intakes were similar between treatments, which agreed with Bernard et al. [33], who fed goats with sunflower and linseed and found similar DM intakes. It is important to note that dietary treatments were formulated to be iso-proteic and iso-energetic and, therefore, no differences were expected with regard to nutrient intake.

Calcium soaps of CO were more digestible compared with SO and PO (Table 3). Several studies have mentioned that the use of calcium soaps does not have any adverse effect on dry matter digestibility [34,35]. Although dietary treatments in this study had similar chemical composition, it may be possible that the fiber contents (NDF and ADF) from SO and PO affected fibrolytic bacteria. However, this contention warrants further investigation. In this study, canola oil had around 65% oleic acid, which is a monounsaturated FA and is therefore less toxic to some of the ruminal microorganisms, which may be the reason for the higher digestibility shown in the CO treatment [36,37].

### 3.3. Nitrogen Balance

No differences were observed for N intake and excretion (Table 4). The lack of differences in N utilization attests to the uniformity of energy and crude protein contents of dietary treatments. Dietary energy is one of the most important factors to consider in ruminant diets as it has a direct impact on microorganisms and overall protein metabolism at the rumen level [38]. Therefore, if dietary treatments were iso-energetic, no changes to N utilization were expected. The animals fed with CO and PO treatments were found in positive nitrogen balance, and only with SO treatment were we able to observe a numerical N loss of 0.03 g/kg LW. In ruminants, it has been reported that dietary FA affects microbial protein synthesis, protozoa counts and methanogen profile [39], which can contribute to improving the efficiency of energy utilization of the feeding [4]. However, no differences in N balance were found between treatments in this study. The positive N balance found with CO and PO diets indicated the capacity of the diets to supply the N required for the goat’s maintenance, without the need for energy mobilization from body reserves, and while also supplying adequate amounts of protein to rumen microorganisms [19].

### 3.4. Milk Production and Milk Composition

Milk production (Table 5) was higher in SO compared with CO and PO. Mir et al. [40] fed dairy goats with four inclusion levels of canola oil without observing any effect on milk production. Similarly, Chilliard et al. [41] and Lock et al. [42] reported that the supplementation of fats in dairy goat diets had no effect on milk production. In contrast, Lu [43] found a decrease in milk production with the supplementation of animal fat at 5% DM in lactating goats. In order to have more accurate inferences for milk production, milk production efficiency, corrected by fat (fat-corrected milk 3.5%), energy (energy-corrected milk) and feed efficiency (feed efficiency and fat-corrected milk 3.5%), were calculated, and similar results were obtained between treatments. This was a unique feature of the study, as the calculations allowed us to understand milk production from different angles. Thus, it appeared that SO was the best for milk yield, but if we accounted for energy utilization from the diet, all treatments resulted in similar values.

Palmquist [44] reported that fat supplementation increases energetic efficiency in lactating cows by increasing total energy intake by generating ATP more efficiently compared with volatile fatty acids, and by directly incorporating long-chain FA into milk fat. Also, one mechanism proposed for the increase in milk yield with fat supplementation is glucose sparing, in which the suppression of de novo FA synthesis in the mammary gland decreases the oxidative use of glucose to generate reducing equivalents for milk fat synthesis [45]. This extra glucose may be utilized in other milk processes, including lactose synthesis and increased milk yield [46].

Contrary to cow research, some articles have mentioned that the use of fat supplements does not change milk production in dairy goats [32,41]. With regard to fat and protein content in milk, CO was lower than SF and PO. Although dietary lipid supplementation in dairy goats generally improves milk fat production [32], in this study, this was not the case for CO treatment, which resulted in the lowest amount of fat. It has been reported that the effect of supplemental fats on milk fat is not always positive since it is influenced by their percentage of inclusion in the diet, type of forages in the diet, type of supplemental fat (rumen-protected or not), physical form (i.e., whole seeds and oils) and the chemical composition of dietary lipids (saturated or unsaturated fatty acids) [41].

**Figure 1:**
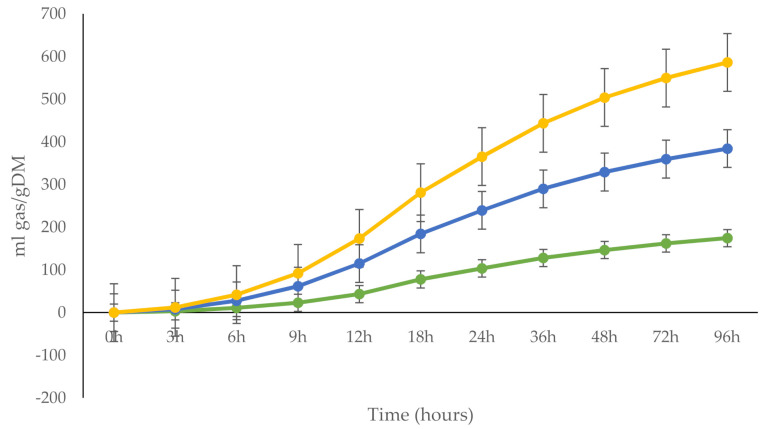
Total gas production (mL gas/g DM) over 96 h of in vitro incubation from calcium soaps of palm oil (yellow line), safflower oil (blue line) and canola oil (green line).

It is worth mentioning that CO resulted in inferior ruminal fermentation performance, as shown by a reduction in total gas production (Figure 1) and a reduction in the amount of SCFA, which could have influenced the lower amount of milk fat found for this treatment. This is important since SCFAs, such as acetate and butyric acids, play an important role in the formation of short-chain fatty acids (C4:0 to C14:0) in milk. These fatty acids account for approximately 60 and 45% of the total milk fatty acids on a molar and weight basis, respectively [47].

Non-fat solids were lower in CO and PO compared with SO, and total solids were higher for SO compared with PO and CO. The addition of fat leads to a reduction in rumen fermentable organic matter, reduction in precursors of glucose and reduction in the synthesis of microbial protein, and thus a reduction in the amount of amino acids available for the synthesis protein in milk. This explains why a lower amount of protein in milk was observed with regard to the fat content.

Some studies have reported a reduction of protein in milk in which long-chain FAs were abomasally infused in cows, and showed that the magnitude of changes in milk components depended on the amount and type of FA supplied [48]. Despite the fact that dietary treatments supplied similar protein and energy, the type of FA in each calcium soap elicited differential effects on protein contents in milk, and in this case, CO resulted in a detrimental effect on this milk component.

For lactose content in milk, no differences were observed among treatments (*p* > 0.05); however, Luna et al. [49] reported differences in goat milk lactose when the diets were supplemented with whole linseed (1.84% DM) and sunflower oil (0.81% DM). Ayeb et al. [50] found no differences in the lactose content of goat milk when animals were fed dry olive leaves ad libitum. These authors reported an increase of the total solids when dry olive leaves were included in the diet.

### 3.5. In Vitro Gas Production

The parameters of the in vitro gas production of the ingredients used in the diets are presented in Table S1. Differences in gas production for each ingredient (*p* < 0.001) were observed, being higher (*p* < 0.001) in corn silage and corn grain, and lower in PO. The fractional rate of degradation (c) and lag time was similar among ingredients (*p* > 0.1), DMD 96 h was higher (*p* < 0.001) for soybean and corn grain, followed by barley hay and corn silage, with DMD being lower for safflower oil, canola oil and palm oil. PG 96 h was higher (*p* < 0.05) for corn grain than for the other ingredients. Gas production is an indirect measure of the degradation of substrates, particularly from carbohydrates. Furthermore, it is an estimator for the production of short-chain fatty acids [30,51]. GY 24 h, SCFA and MCP synthesis was higher (*p* < 0.001) for soybean, corn grain and barley hay compared to SO, CO and PO (Figure S1). The results obtained mirrored the chemical composition of each feedstuff, which could increase or decrease microbial fermentation in addition to the fact that SO, PO and CO are protected fats, and protected fats in the form of calcium soaps usually depress gas production [31].

The in vitro gas production parameters were different between treatments (*p* < 0.002) (Table 6). Increased gas production (mL gas/g DM) and the “c” fraction were higher for SO (*p* < 0.001) followed by PO, where the inclusion of CO affected the gas production and fermentation rate. Lag time was higher (*p* < 0.001) for CO than SO. Fat, oils and grease have negative effects on rumen fermentation, which are associated with the inhibition of microbial activity, particularly microorganism methanogenic activity [52]. It has been mentioned for many years that oil supplementation, particularly with unsaturated oils, affects and modifies the microbial activity of the rumen, which is involved in cellulose degradation. Also, it has been explained that these factors are due to the fact that fats coat the ruminal bacteria, disrupting cell membrane functions, and that long-chain fatty acids are toxic to cellulolytic bacteria [53].

In the present study, less gas production was observed in the treatment of CO, followed by SO, which are unsaturated fatty acids such as linoleic acid. As mentioned above, this type of fatty acid is toxic, causing fewer protozoa and bacteria in the rumen, which explains our findings. The DMD 96 h, PF 96 h and MCP were not affected among treatments (*p* > 0.1). GY 24 h and SCFA were higher (*p* < 0.001) for SO and PO than CO. Under the conditions of this study, results from in vitro gas production pointed at the fact that there were differential effects on rumen fermentation based on the degree of FA saturation as palm oil was a saturated FA source, CO was mainly a monounsaturated FA and SO was primarily formed by polyunsaturated FA. In this study, treatments exerted different magnitudes of change that, to some degree, led to the inhibition of ruminal fermentation processes and affected carbohydrate digestion [54]. Overall, the discrepancies between the higher digestibility detected with CO and its lower gas production remain unknown, and perhaps analyzing the rumen microbiome will clarify these findings.

Taken together, our study confirms pioneer studies (i.e., [17]) reporting that calcium soaps are an effective source of fat for dairy rations because ruminal fermentation is normal or at least not negatively affected, digestibility of fatty acids is high and soaps are mixed easily with other feed ingredients (as observed when diets and treatments are mixed and supplied).

## 4. Conclusions

Overall, compared with the traditional use of calcium soaps manufactured from PO, at an inclusion of 2.7% DM, calcium soaps of SO can be used in goat diets to increase milk production, milk protein and milk fat yields without negative effects on nutrient intake and digestibility. Further studies should consider the analysis of milk fatty acid profiles to evaluate the transfer of dietary fatty acids supplied into milk fat and rumen microbiome analysis warrants further attention in order to improve our understanding on nutrient digestibility and in vitro gas production.

## Figures and Tables

**Table 1:** Chemical composition (g/kg dry matter (DM)) of individual feedstuffs and lipid supplements used in the experimental diets.

Ingredient ^1^	DM	CP	EE	NDF	ADF	Ca	P	ME ^3^
Soybean meal	900	440	17	161	80	3	7	12.9
Barley hay	880	120	20	650	370	1	4	8.8
Corn grain	900	80	41	145	26	1	3	14.5
Corn silage	387	68	30	550	300	2	2	10.9
Calcium soaps of palm oil	972	0	848	0	0	18	0	26.8
Calcium soaps of canola oil	969	0	840	0	0	18	0	25.8
Calcium soaps of safflower oil	970	0	845	0	0	18	0	26.4
Vitamin and mineral premix ^2^	1000	0	0	0	0	4.5	0.14	0

^1^ DM = Dry matter; CP = crude protein; EE = ether extract; NDF = neutral detergent fiber; ADF = acid detergent fiber; ME = metabolizable energy (MJ/kg DM). ^2^ Containing in 1.0 kg DM the following: 25 mg of antioxidant, 4.5 g of calcium carbonate, 6 g of salt, 30 g of ionophore, 50 g of zinc oxide, 6 g of sodium bicarbonate, 6 g of copper sulphate, 20 g of ferrous sulphate, 125 g of sodium sulphate, 18,000 IU of vitamin E, 3,000,000 UI of vitamin A, 3,750,000 IU of vitamin D, 140 g of potassium chloride, 0.500 g of EDD. I ethylene-dynamine, 0.090 g of cobalt carbonate, 500 mg of magnesium oxide, 36 g of manganese oxide and 0.090 g of selenium. ^3^ Calculated from the National Research Council [19].

**Table 2:** Ingredients and chemical composition of safflower oil (SO), canola oil (CO) and palm oil (PO) treatments in dairy goat diets.

Parameters	SO	CO	PO
Ingredients, g/kg DM			
Corn silage	500	500	500
Soybean meal	102	102	102
Barley hay	105	105	105
Corn grain	250	250	250
Calcium soaps of palm oil	0	0	27
Calcium soaps of canola oil	0	27	0
Calcium soaps of safflower oil	27	0	0
Vitamin and minerals premix ^1^	16	16	16
Chemical composition, g/kg DM			
Dry matter	644	644	644
Crude protein	111	111	111
Neutral detergent fiber	395	395	395
Acid detergent fiber	203	203	203
Ether extract	40.6	40.7	40.9
Ca	2.1	2.2	2.2
P	2.9	2.9	2.9
Metabolizable energy, MJ/kg DM ^2^	12.0	12.0	12.0

^1^ Containing in 1.0 kg DM the following: 25 mg of antioxidant, 4.5 g of calcium carbonate, 6 g of salt, 30 g of ionophore, 50 g of zinc oxide, 6 g of sodium bicarbonate, 6 g of copper sulphate, 20 g of ferrous sulphate, 125 g of sodium sulphate, 18,000 IU of vitamin E, 3,000,000 UI of vitamin A, 3,750,000 IU of vitamin D, 140 g of potassium chloride, 0.500 g of EDD. I ethylene-dynamine, 0.090 g of cobalt carbonate, 500 mg of magnesium oxide, 36 g of manganese oxide and 0.090 g of selenium. ^2^ Calculated from NRC [19].

**Table 3:** Nutrient intake and digestibility of goats supplemented with safflower oil (SO), canola oil (CO) and palm oil (PO) treatments ^1^.

Item	SO	CO	PO	SEM	*p*-Value
Initial live weight, LW, kg	52.0	51.1	51.6	1.64	0.938
Final live weight, LW, kg	52.6	51.3	54.6	1.76	0.453
Average live weight, LW, kg	52.3	51.2	53.1	1.37	0.635
Average metabolic live weight, LW^0.75^	19.4	19.1	19.6	0.38	0.637
Intake, g/d					
Dry matter	1820	1968	2020	175	0.716
Organic matter	1667	1765	1810	157	0.812
Neutral detergent fiber	720	779	800	69.1	0.716
Acid detergent fiber	370	400	411	35.5	0.717
Fat	74	80	82	7.11	0.689
Metabolizable energy, MJ/d ^2^	20	21	22	1.90	0.803
Intake, g/kg LW^0.75^					
Dry matter	92.9	102	105	10.2	0.709
Organic matter	85.1	91.7	93.7	9.21	0.795
Neutral detergent fiber	36.8	40.5	93.4	4.05	0.709
Acid detergent fiber	18.9	20.8	21.3	2.08	0.709
Fat	3.8	4.2	4.3	0.42	0.686
Metabolizable energy, MJ/d ^2^	1.02	1.10	1.13	0.11	0.795
Digestibility, g/kg					
Dry matter	552 ^b^	653 ^a^	588 ^b^	15.5	0.001
Organic matter	559 ^b^	663 ^a^	606 ^ab^	17.6	0.001
Neutral detergent fiber	460 ^b^	616 ^a^	510 ^b^	17.5	0.001
Acid detergent fiber	424 ^c^	658 ^a^	519 ^b^	16.9	0.001

^a–c^ Different literals within the same column show significant differences (*p* < 0.05). SEM = standard error mean. ^1^ Treatments were calcium soaps of safflower oil (SO), canola oil (CO) and palm oil (PO, Megalac-R^®^, Arm & Hammer Animal Nutrition, Princenton, NJ, USA), supplemented at 2.7% DM; ^2^ Calculated from NRC [14].

**Table 4:** Nitrogen balance (g/d, g/kg LW^0.75^) in dairy goats fed with safflower oil (SO), canola oil (CO) and palm oil (PO) treatments ^1^.

Item	SO	CO	PO	SEM	*p*-Value
g/d					
N intake	32.46	35.09	36.04	3.11	0.716
N excretion					
Urine	11.79	12.87	13.84	2.61	0.860
Feces	21.18	12.52	16.40	3.30	0.257
N Balance	−0.51	9.69	5.79	4.41	0.326
g/kg LW^0.75^					
N intake	1.65	1.82	1.86	0.18	0.709
N excretion					
Urine	0.60	0.66	0.71	0.13	0.852
Feces	1.08	0.64	0.85	0.17	0.294
N Balance	−0.03	0.51	0.30	0.23	0.323

^1^ Treatments were calcium soaps of safflower oil (SO), canola oil (CO) and palm oil (PO, Megalac-R^®^, Arm & Hammer Animal Nutrition, Princenton, NJ, USA), supplemented at 2.7% DM.

**Table 5:** Milk yield (kg/d) and milk composition from dairy goats supplemented with safflower oil (SO), canola oil (CO) and palm oil (PO) treatments ^1^.

Item	SO	CO	PO	SEM	*p*-Value
Milk production, kg/d	1.10 ^a^	0.95 ^b^	0.88 ^b^	0.09	0.009
Fat-corrected milk 3.5%	1.07	0.93	0.88	0.18	0.740
Energy corrected milk	1.08	0.99	0.86	0.17	0.667
Feed efficiency	0.36	0.30	0.24	0.05	0.169
FE FCM	0.36	0.30	0.25	0.05	0.265
Milk production efficiency	6.19	6.59	4.80	0.88	0.330
Fat, g/d	35.0 ^a^	32.3 ^b^	34.3 ^a^	0.56	0.002
Protein, g/d	37.7 ^a^	29.8 ^b^	34.6 ^a^	0.48	0.003
Lactose, g/d	45.1	39.9	37.0	3.82	0.076
Total solids, g/d	127 ^a^	111 ^b^	113 ^b^	0.85	0.005
Non-fat solids, g/d	100 ^a^	78.9 ^b^	78.9 ^b^	0.96	0.002
Fat, g/kg	33 ^b^	35 ^b^	41 ^a^	0.52	0.001
Protein, g/kg	35 ^b^	33 ^b^	41 ^a^	0.57	0.003
Lactose, g/kg	41	42	42	0.85	0.365
Total solids, g/kg	120 ^b^	122 ^b^	136 ^a^	1.75	0.049
Non-fat solids, g/kg	95 ^a^	86 ^b^	94 ^a^	0.91	0.002

^a,b^ Different literals within the same column show significant differences (*p* < 0.05). SEM = standard error mean. NFS = nonfat solids. Fat-corrected milk (FCM 3.5%, kg/d) = [milk (kg/d) × 0.432] + [fat (kg/d) × 16.216], energy corrected milk (ECM) = [milk (kg/d) × 0.327] + [fat (kg/d) × 12.86] + [protein (kg/d) × 7.65] [24], feed efficiency (FE) = milk yield (kg/d)/dry matter intake (kg/d), feed efficiency corrected (FE FCM) = milk yield (FCM, 3.5%, kg/d)/dry matter intake (kg/d), MPE = milk production efficiency (kg of milk produced/kg of crude protein ingested). ^1^ Treatments were calcium soaps of safflower oil (SO), canola oil (CO) and palm oil (PO, Megalac-R^®^, Arm & Hammer Animal Nutrition, Princenton, NJ, USA), supplemented at 2.7% DM.

**Table 6:** *In vitro* rumen gas kinetics, cumulative gas production and rumen fermentation profile, after 96 h of incubation in goat diets supplemented with safflower oil (SO), canola oil (CO) and palm oil (PO) treatments ^1^.

Item	SO	CO	PO	SEM	*p*-Value
B	206 ^d^	174 ^e^	201 ^d^	4.6	<0.001
C	0.05 ^d^	0.04 ^e^	0.04 ^de^	0.001	0.001
Lag time	4.47 ^e^	5.23 ^d^	4.86 ^de^	0.188	0.030
Gas production, mL gas/g DM					
6 h	16.6 ^a^	11.0 ^b^	14.3 ^ab^	1.42	0.033
12 h	71.3 ^a^	43.5 ^b^	58.5 ^a^	3.56	<0.001
24 h	135 ^a^	103 ^b^	125 ^a^	4.59	<0.001
48 h	182 ^a^	146 ^b^	174 ^a^	4.97	<0.001
DMD 96 h	74	72	77	5.6	0.782
PF 96 h	290	293	263	34.8	0.796
GY 24 h	33.9 ^a^	25.9 ^b^	31.4 ^a^	1.14	<0.001
SCFA	0.75 ^a^	0.57 ^b^	0.69 ^a^	0.03	<0.001
MCP	670	660	704	57.6	0.854

^a,b,d,e^ Mean values in the same row with different literals are statistically different. SEM = standard error of the mean. ^1^ Treatments were calcium soaps of safflower oil (SO), canola oil (CO) and palm oil (PO; Megalac-R^®^, Arm & Hammer Animal Nutrition, Princenton, NJ, USA). B = fermentation rate (h^−1^), C = fermentation rate (h^−1/2^), lag time = time in which fermentation starts, DMD 96 h = dry matter disappeared at 96 h (mg/100 mg); PF 96 = partition factor (mL gas/g DMD 96 h); GY 24 h = gas production at 24 h (mL gas/g DM), SCFA = short-chain fatty acids (mL/200 mg DM), MCP = microbial protein (mg/g DM).

## Supplementary Materials

The following are available online at https://zenodo.org/record/3928288#.X2iWoWgzZaS, Figure S1: Total gas production (mL gas/g DM) from feedstuffs used for the experiment: corn grain cracked*; soybean meal, ⬤; barley hay, ◆; canola oil, ⬛; palm oil, ▲; safflower oil, ⬤; corn silage, ▲. Table S1: *In vitro* rumen gas kinetics, cumulative gas production and rumen fermentation profile after 96 h of incubation as affected by the ingredients used in goats with different calcium soaps sources.
